# Chemical composition of biomass generated in the guava tree pruning

**DOI:** 10.17179/excli2014-467

**Published:** 2015-02-04

**Authors:** Julio César Camarena-Tello, Nuria Elizabeth Rocha-Guzmán, José Alberto Gallegos-Infante, Rubén Francisco González-Laredo, Fabiola Eugenia Pedraza-Bucio, Pablo López-Albarrán, Rafael Herrera-Bucio, José Guadalupe Rutiaga-Quiñones

**Affiliations:** 1 Facultad de Ingeniería en Tecnología de la Madera, Edificio D, Ciudad Universitaria, Universidad Michoacana de San Nicolás de Hidalgo, Av. Fco. J. Múgica S/N. Col. Felicitas de Río, Morelia, Michoacán, C.P. 58040, México; 2 Departamento de Ingenierías Química y Bioquímica, Instituto Tecnológico de Durango, Blvd. Felipe Pescador 1830 Ote., Col. Nueva Vizcaya, Durango, Durango, C.P. 34080, México

**Keywords:** Psidium guajava, pH, ash, extractives, polysaccharide, tannins

## Abstract

*Psidium guajava* L. (*Myrtaceae*) is a native plant of Central America and is now widely cultivated in many tropical regions of the world for the fruit production. In Mexico, in the guava orchards common practices to control fruit production are: water stress, defoliation and pruning. In this study, we report the chemical composition of the biomass (branches and leaves) generated in the pruning practices. The results ranged as follows: pH (4.98-5.88), soda solubility (39.01-70.49 %), ash (1.87-8.20 %); potassium and calcium were the major inorganic elements in ash. No heavy metals were detected in the studied samples; total solubility (15.21-46.60 %), Runkel lignin (17.77-35.26 %), holocellulose (26.56 -69.49 %), α-cellulose (15.53-35.36 %), hemicelluloses (11.02-34.12 %), tannins in aqueous extracts (3.81-9.06 %), and tannins in ethanolic extracts (3.42-15.24 %).

## Introduction

*Psidium guajava* L. belongs to the *Myrtaceae* family. It is a native of Central America but is now widely cultivated, distributed and the fruits enrich the diets of millions of people in the world tropics (Rathish and Sumitra, 2007[29]; El-Mahmood, 2009[14]). In Mexico, the States with the largest fruit production are Michoacan (42 %), Aguascalientes (35 %) and Zacatecas (15 %), the rest (8 %) belongs to other states (González Gaona et al., 2002[16]). In Michoacan, the maximum fruit production falls into three municipalities located in the eastern area of the state: Jungapeo (2,500 hectares), Benito Juárez (1,500 hectares) and Zitácuaro (1,000 hectares) (Mendoza Lopez et al., 2005[22]). In these guava orchards common practices to control fruit production are: water stress, defoliation and pruning; pruning is the most used activity. 

*P. guajava *is a well known traditional medicinal plant used in some indigenous systems throughout the world. All parts of this tree, including roots, bark, leaves, seeds, and the fruits have been used for treatment gastrointestinal problems. Leaves, pulp and seeds are used as an antispasdomic, anti-inflammatory, and anti-diarrheic, to treat respiratory and gastrointestinal disorders, in the treatment of hypertension, obesity and in the control of diabetes mellitus (Barbalho et al., 2012[5]). It also possesses anticancer properties (Ryu et al., 2012[33]). The seeds of *P. guajava* are also used because of their antimicrobial, gastrointestinal, anti-allergic, and anti-carcinogenic activities (Pelegrini et al., 2008[28]; Metwally et al., 2010[23]; Huang et al., 2011[19]; Bontempo et al., 2012[8]). The leaves are used in the form of poultice or decoction for wounds, ulcers and toothaches, they are also used for treatment bronchitis, asthma attacks, cough, and pulmonary diseases (Shruthi et al., 2013[35]). Stem-bark extracts can be used to treat malaria (Nundkumar and Ojewole, 2002[25]). The bark is used as an astringent in the treatment of ulcers wounds, diarrhea, dysentery, skin ailments, vaginal hemorrhage wounds, fever, dehydration, and respiratory disturbances; the root is used as a decoction to treat diarrhea, coughs, stomach ache, indigestion, toothaches, and constipation; the whole plant is in general used in the form of decoction, infusion and paste as skin tonic (Gutiérrez et al., 2008[17]). 

Many biological works have been carried out on different parts of the *P. guajava* tree. Particulary, in this study we report the chemical composition of the biomass (branches and leaves) generated in the pruning, which usually causes problems and promotes proliferation of pests in orchards. In guava orchards in Mexico, a guava tree can produce about 9 kg of foliar biomass in a year (Damián Nava et al., 2004[12]) and the density of trees per hectare ranges from 154 to 666 (González Gaona et al., 2002[16]) so it can generate 1,386 to 5,994 kg of dry biomass per hectare in a year, which could be exploited. 

## Materials and Methods

### Biomass from pruning

For this work, four sites in Michoacan, Mexico were chosen because they represent the largest areas of guava tree plantations with the higher volumes of fruit exportation. The four selected sites are located at different elevations above sea level, chosen an orchard from each place. Harvesting of the biomass (branches and leaves) was performed from 14^th^ to 16^th^ August 2013 at the four sites: 1) “Hichachico”, Municipality of Nuevo Urecho located at 19°12'26.37' Lat. N. and 101°52’35.06’’ Long. W., at 533 meters above sea level (masl); 2) “La Vega”, Municipality of Jungapeo located at 19°27’21.52’’ Lat. N. and 100°30’4.22’’ Long. W., at 1,251 masl; 3) “El Cerrito”, Municipality of Jungapeo located at 19°28’38.51’’ Lat. N. and 100°28’28.43’’ Long. W., at 1,659 masl; 4) “Paraje Guarda Ganado”, Ejido de Zirahuato located at 19°29’59.09’’ Lat. N. and 100°25’5.96’’ Long. W., at 2,030 masl. Thirty trees were sampled randomly in each orchard and every tree pruning biomass was collected. The pruning to collect biomass is usually performed after the guava fruit harvesting is done. Moisture content in branches and leaves was determined immediately after the pruning with the dehydration method at 105 ± 3 °C, according to the Technical Association for Pulp and Paper Industry (TAPPI) standard procedure, T 264 cm-97 (TAPPI, 2000), then the net amount of biomass produced per tree was calculated. The branches and leaves were separated from the biomass collected and allowed to dry in the shade. 

Separately, branches and leaves were milled with a laboratory mill (Mikron K20), and then, sieved with 40-fraction mesh (425 micron pores) for chemical analysis. Subsequently, moisture content was determined with the dehydration method at 105 ± 3 °C, according with the TAPPI standard, T 264 cm-97 (TAPPI, 2000[36]). Wood meal 40 mesh is stored in dark sealed containers until use. All determinations were performed in duplicate. 

### Chemical analysis

Determinations of pH were based on a method described by Sandermann and Rothkamm (1959[34]). Sosa solubility was determined by TAPPI standard, T 212 om-98 (TAPPI, 2000[36]). Mineral content was calculated gravimetrically in accordance with the TAPPI standard, T 211 om-93 (TAPPI, 2000[36]). Microanalysis of the ash was carried out with an X-ray spectrometer fitted to a Jeol-brand scanning electron microscope (Model JSM-6400) five times and the operating conditions for the analysis were 20 kV and 8.5 s (Téllez-Sánchez et al., 2010[37]). To determine the total content of extractives, sequential extractions were performed with a Soxhlet apparatus using the following solvents: cyclohexane, acetone, methanol, and finally, hot water under reflux (6 h in each case). Solvents were recovered in a rotary evaporator and the respective extracts were stored in a dessicator until the weight was constant. After sequential extractions, wood meal intended as an extractive-free wood meal, was used to determine Runkel lignin (Runkel and Wilke, 1951[32]) and holocellulose (Wise et al, 1946[39]). The a-cellulose content was based on an American Society for Testing and Materials (ASTM) standard, D 1104-57 (ASTM, 2000[3]), where chlorine gas was replaced with sodium hypochlorite. Hemicelluloses were determined by difference between holocellulose and α-cellulose. 

### Tannins content

In the original material, tannin content was also determined (Yazaki and Hillis, 1977[43]; Waterman and Mole, 1994[38]); for this purpose aqueous and ethanol extractions were performed. 

## Results and Discussion

### Chemical analysis

After collection of biomass, the average moisture content in branches was 68 % (± 2.63 %) and in leaves 75 % (± 2.98 %). The average result of biomass generated by the pruning in a tree was 12 kg. This value is slightly higher than that reported for the generation of foliar biomass (Damián Nava et al., 2004[12]). 

**Figure 1:**
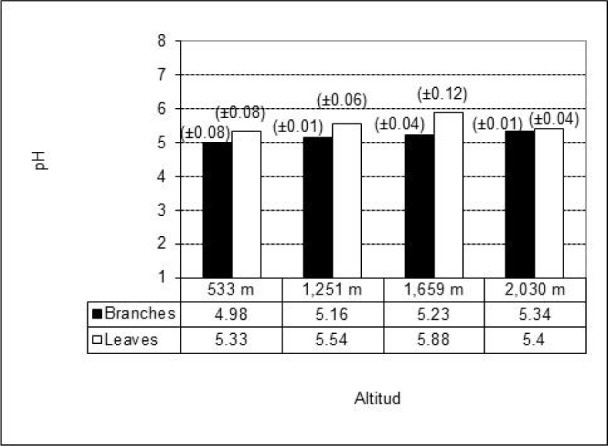
pH values in the biomass samples

The average measurements and standard deviations of the chemical components concentrations are presented in Table 1(Tab. 1). The results from the X-ray microanalysis of ash are shown in Table 2(Tab. 2). The pH values for the studied material obtained from thirty trees ranged from 4.98 to 5.88 and they were weakly acid (Kollmann, 1959[20]). The variation in wood pH is associated to the presence of acid groups, free acid, to the amount and type of extractives and to climatic factors (Fengel and Wegener, 1989[15]). The material from the branches (wood and bark) was slightly more acidic (average value of 5.18) than the material from the leaves (average value of 5.54) (Standard deviation in parenthesis, Figure 1(Fig. 1)). Information about the pH of branches and leaves of *P. guajava* were not available, but the pH values in branches were within the range for woods from temperate zones (Fengel and Wegener, 1989[15]). 

**Figure 2:**
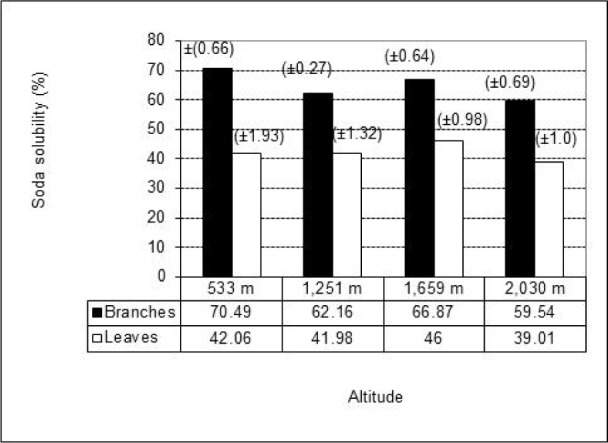
Soda solubility in the biomass samples

The results of one percent sodium hydroxide solubility for the studied material obtained from thirty trees ranged from 39.01 to 70.49 % and the highest values corresponded to branches (Standard deviation in parenthesis, Figure 2(Fig. 2)) with an average value of 64.76 % compared to the average value of 42.26 % in leaves. The results found here are higher than those reported for some pine woods (17.9-25.4 %) (Bernabé-Santiago et al., 2013[6]). It is known that a hot alkali solution can extract low-molecular-weight carbohydrates mainly of hemicellulose and the soda solubility of lignocellulosic materials could indicate its natural durability (T 212 om-98, TAPPI, 2000[36]) so it is expected that this biomass from *P. guajava* can be easily damaged by the action of microorganisms, heat and sun light. 

The ash content values ranged from 1.87 to 8.20 % and it was clear that the leaves are richer in minerals than branches. From these results it appears that at a higher altitude is higher the ash content. The mineral contents in branches are higher than the reported 1.21 % by Hindi (2012[18]), and 2.5 % by Luiz Adrian et al. (2012[21]). However, our results are in the range reported for tropical wood species, which are richer in ash than woods of temperate zones (Fengel and Wegener, 1989[15]). The ash content values for leaves are higher than the value reported for guava leaves (Okunrobo et al., 2010[26]).

Regarding the microanalysis of ashes, a variation was observed in the number of detected chemical elements: in branches from the orchard located at 1,659 masl six chemical elements were detected, while in the leaves from the trees collected in the orchard located at 533 masl nine chemical elements were found (Table 2(Tab. 2)). The major inorganic compounds in ash were potassium and calcium in all samples. The potassium concentration in the branches was higher than in leaves. Most of the chemical elements detected in the branches have been previously reported in other wood and barks (Cutter et al., 1980[11]; Fengel and Wegener, 1989[15]). Some chemical elements detected here in leaves have also been reported in leaves of guava (Mubarak et al., 2008[24]; Okunrobo et al., 2010[26]; Dhiman et al., 2011[13]). An important fact is that no heavy metals were detected in the studied samples. The pruning biomass from *P. guajava*, which is rich in inorganic substances is attractive to be used as compost, since their minerals are important for plants (Restrepo-Rivera and Pinheiro, 2009[30]). 

The results from successive extractions vary with the solvent used and extracted samples (Table 1(Tab. 1)). The total solubility ranged from 15.21 to 46.60 % and it clearly shows that the leaves are richer in the extractive compared to the branches. The lowest extractives content (0.22 %) was observed in branches, using cyclohexane, and the highest (21.3 %) in leaves using hot water. In general, the high amount of extractives in all samples was observed using hot water and methanol as solvents, which could confirm the high polyphenol and carbohydrates content in these samples, as these substances can be extracted with such solvents (Fengel and Wegener, 1989[15]). Lipophilic substances are scarce in branches samples, except in leaves (1.97-2.85 %). Hindi (2012[18]) found 12.6 % total extractives in branches of* P. guajava* and this value is lower compared with the lowest yield reported here for branches. 

The Runkel lignin content in branches ranged from 17.87 to 19.54 %, lower values compared to that found (48 %) by Avelar Mejía et al. (2003[4]) and (27.1 %) by Hindi (2012[18]). In the case of the leaves, the lignin content ranged from 17.77 to 35.26 % and clearly showed that the leaves are richer in lignin compared to the branches. Because the leaves are rich in lignin, these biomass generated from pruning may be used to make pellets, or possibly enrich any lignocellulosic based material; it is known that lignin has a high calorific value (Kollmann, 1959[20]; Browning, 1963[9]). 

Regarding to the polysaccharides content of the guava pruning biomass, the holocellulose values ranged from 26.56 to 69.49 % and the cellulose values ranged from 15.53 to 35.36 %, which are within the range of values determined for others lignocellulosic materials: holocellulose in hardwoods (49.2 – 89.2 %), cellulose in hardwoods (37.6 – 56.2 %) (Fengel and Wegener, 1989[15]). 

### Tannins content

The results of tannins in biomass of *P. guajava* are summarized in Table 3(Tab. 3) and Table 4(Tab. 4). The values for total extract using water as solvent ranged from 7.65 % in branches to 20.95 % in leaves and using ethanol from 7.32 % in branches to 22.48 % in leaves. It clearly shows that the total extract content is higher in leaves compared with branches. These values are slightly higher than those reported for other lignocelullosic materials (Rosales Castro and González Gaona, 2003[31]; Colín-Urieta et al., 2013[10]). 

The Stiasny number ranged for water extracts from 36.88 % in leaves to 56.97 % in branches of guava and for the ethanolic extracts from 39.02 % in branches to 67.80 % in leaves. The Stiasny numbers were higher in leaves compared with values obtained in branches. The Stiasny number indicates a relatively high content of proantocyanidins or condensed tannins reagents (Wissing, 1955[40]), which may be reacted with formaldehyde in acidic medium (Yazaki and Hillis, 1980[42]; Yazaki et al., 1993[41]). In general, the results of Stiasny number reported for others lignocellulosic materials (Rosales Castro and González Gaona, 2003[31]; Colín-Urieta et al., 2013[10]) are in the range for the values obtained in this work.

Regarding the tannins content by the water extraction, it ranged from 3.81 % in branches to 9.06 % in leaves, while using ethanol as solvent the values ranged from 3.42 % in branches to 15.24 % in leaves. These results indicate higher concentration of tannins in the leaves compared to the branches. The tannins amount reported for others lignocellulosic materials (Rosales Castro and González Gaona, 2003[31]; Pedraza-Bucio and Rutiaga-Quiñones, 2011[27]; Colín-Urieta et al., 2013[10]) are in the range for the values obtained in this work. The presence of tannins in ethanolic extracts of leaves of* P. guajava* has been also reported in others studies (Akinjogunla et al., 2010[2], 2011[1]; Biswas et al., 2013[7]). 

## Conclusions

Branches and leaves as biomass generated in the guava tree pruning collected in four different sites were characterized. The pH values for the studied biomass were weakly acidic. The concentration of inorganic material is high, particularly in the leaves. The major inorganic substances in ash were potassium and calcium in all samples. No heavy metals were found in branches and leaves. The guava tree materials have high solubility in soda, particularly the branches, and high solubility in organic solvents, particularly the leaves. It was found that the lignin and polysaccharide contents are comparable to others lignocellulosic materials. The amount of tannins is higher in leaves compared with branches. It was observed that the chemical composition is not influenced clearly by the altitude of the sites, where the biomass samples were collected. 

## Acknowledgements

The authors thank the Coordination of Scientific Research of the Universidad Michoacana de San Nicolás de Hidalgo, under project No. CIC-21.3-JGRQ, for the financial support. We also thank the producers of guava for donating the study material: Ciriaco Tello Hinojosa, Octavio Valdez Sánchez, Adrián Alvarado and René Jiménez.

This work is dedicated to the memory of Víctor Manuel Rodríguez Alcocer (Universidad Michoacana de San Nicolás de Hidalgo).

## Figures and Tables

**Table 1:**
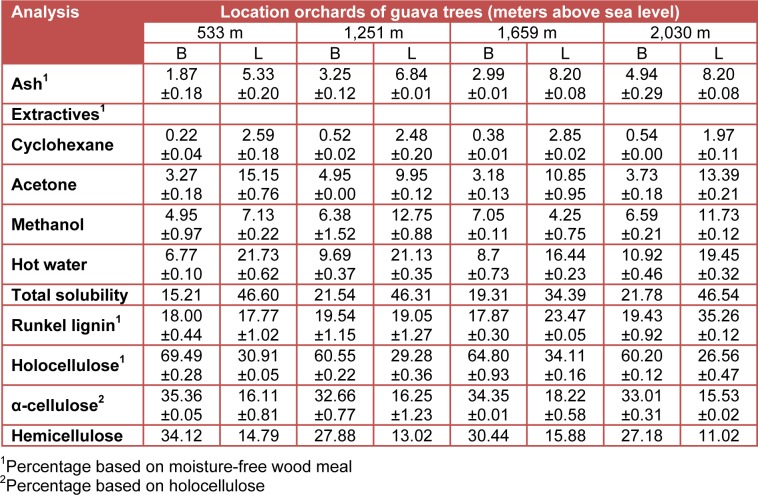
Chemical composition of branches (B) and leaves (L) obtained from thirty trees generated in the *P. guajava* pruning (%)

**Table 2:**
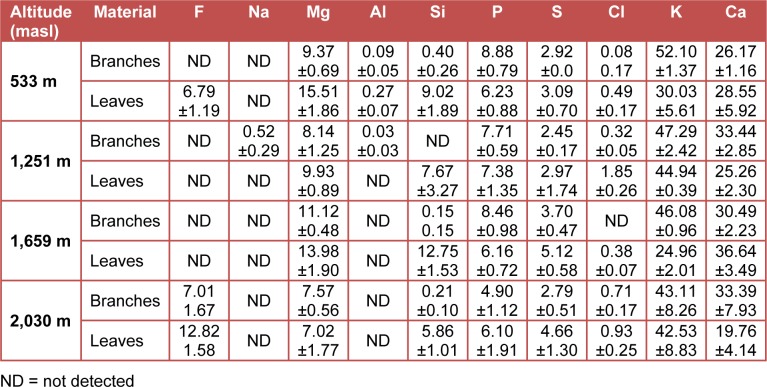
Microanalysis results of biomass of *P. guajava* (%)

**Table 3:**
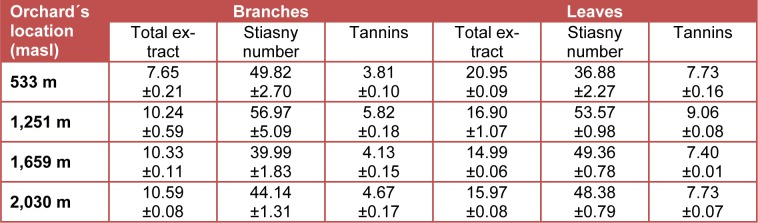
Tannins in the aqueous extract of biomass of *P. guajava* (%)

**Table 4:**
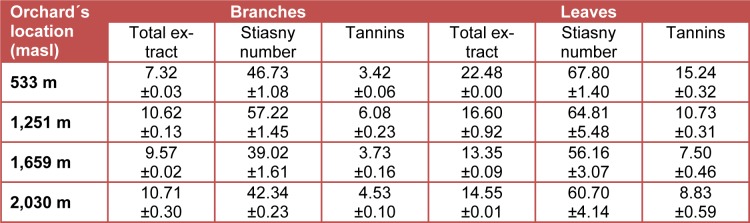
Tannins in the ethanolic extract of biomass of* P. guajava* (%)
